# Optimization of drying conditions for Jackfruit pulp using Refractance Window Drying technology

**DOI:** 10.1002/fsn3.2694

**Published:** 2021-12-30

**Authors:** Sophie Nansereko, John Muyonga, Yusuf B. Byaruhanga

**Affiliations:** ^1^ Department of Food Technology and Nutrition Makerere University Kampala Uganda

**Keywords:** drying temperature, fortification, jackfruit, pulp thickness, response surface methodology

## Abstract

Refractance window drying is a novel technology with high operational efficiency and high product quality retention compared with conventional drying methods. This study assessed the effect of refractance window dryer water temperature and pulp thickness on nutrient content and the antioxidant activity of jackfruit. Response surface methodology (RSM) was used to optimize the drying temperature and fruit pulp thickness. Optimal drying temperature and pulp thickness were found to be 93.4°C and 2.56 mm, respectively. The respective values for the response variables drying time (min), ascorbic acid (mg/100 g), antioxidant activity (mg/100 g AA equiv) and total carotenoid content (μg/g) were 60.47, 17.97, 82.34, and 13.34, respectively. Models for prediction of these values had *R* ^2^ values of .964, .980, .994, and .994, respectively, and nonsignificant lack of fit (*p* < .05). This indicates the suitability of the model in predicting the RWD operating conditions to produce quality dried jackfruit.

## Graphical abstract

In the study, the effect of Refractance window dryer water temperature and pulp thickness on nutrient content and antioxidant activity of jackfruit is assessed and optimized using response surface methodology.

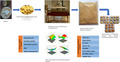

**Novelty Impact Statement**

- Refractance window technology is suitable for drying jackfruit puree for leather
- production.
- Jackfruit optimal drying conditions were found to be 93.4°C water temperature and 2.56 mm pulp thickness.
- Antioxidant activity for dried jackfruit was 82.34 mg/100 g ascorbic acid.

**Practical applications**

Results from this study can be applied in the production of jackfruit leather and powder for use as an ingredient in foods, creating market for the fruit and contributing to diversity in food products.

## 1 INTRODUCTION

Jackfruit (*Artocarpus heterophyllus L*.) is a member of the *Moraceae* family. It is considered an underexploited tropical fruit tree (Swami et al., 2016). Jackfruit originated in India and is now widely cultivated in South and South‐East Asia, in the Caribbean and Latin America and some parts of Africa, including Kenya and Uganda (Ranasinghe et al., 2019). Jackfruit is the largest edible fruit with an annual average yield of 50–80 tons of fruits per hectare of land (Balamaze et al., 2019). Jackfruit comprises 25%–35% aril, 7%–12% seed, and 50%–58% nonedible portion (Cruz‐Casillas et al., 2021).

Jackfruits are a good source of vitamins (A, C, thiamine, riboflavin, and niacin) and minerals (calcium, potassium, iron, sodium, and zinc) (Swami et al., 2012). Jackfruit is perishable and cannot be stored for a long time because of its high moisture and sugar contents, making the fruit susceptible to decomposition by microbial and enzymatic activity (Swami et al., 2016). The conversion of jackfruit aril into a dried form would therefore extend its shelf‐life. Jackfruit powder produced from the jackfruit aril can be used as a flavoring ingredient in food products such as ice cream, yogurt, biscuits, and muffins (Swami et al., 2016).

Dehydration of fruit pulp to make leather effectively preserves nutrients and taste for the off‐season (Diamante et al., 2014). In past decades, the main drying processes for preparing fruit leathers were solar drying, drum drying, and cabinet drying with low cost of production (Pua et al., 2010). These conventional drying processes have adverse effects on product quality, attributable to the relatively high temperatures and long duration (Praveen Kumar et al., 2006). Refractance window (RW) drying is a novel drying technique used to preserve valuable nutrients, as drying occurs at relatively low temperatures (Nindo et al., 2003). Compared to freeze‐drying, refractance window drying is another technology known to retain high product quality. RW drying equipment requires 50% less energy and 50%–70% fewer capital expenses for the same drying capacity (Nindo & Tang, 2007). RW dryers are characterized by high evaporation competence of up to 10 kg/m^2^ h^‐1^, which results in a fast drying time (Raghavi et al., 2018; Zotarelli et al., 2015). The drying rate using RWD mainly depends upon the drying temperature and thickness of the pulp (Castoldi et al., 2015).

The physical properties of fruit powders significantly influence fruit powder products' design, optimization, and performance. The physical/powder properties include bulk density, tapped density and solubility, and the flow properties include Carr index, Hausner's ratio, and flowability (Saifullah et al., 2016). Solubility is a crucial representative of product behavior in an aqueous phase and a general criterion for determining the reconstitution quality of a powder (Mahdi et al., 2017). Therefore, the objective of this study was to evaluate the use of RWD in the drying of jackfruit and determine the optimal drying temperature and fruit pulp thickness, characterize the physical properties of the refractance window dried jackfruit powder, and demonstrate its utilization as an ingredient.

## 2 MATERIALS AND METHODS

### 2.1 Experimental design

The i‐optimal design of the Design‐Expert^®^12 statistical software (Stat‐Ease, Inc., Minneapolis, USA) was used to generate the experimental runs. The independent variables included temperature as a continuous variable and puree thickness as a discrete variable. The response factors were drying time, ascorbic acid, antioxidant activity, and total carotenoid content. The limits for the process components (Table 1) were decided based on results from preliminary work. A total of 21 experimental runs were generated, and these are shown in Table 2.

**TABLE 1:** Coded and actual levels of drying temperature and puree thickness used in the generation of experimental runs

Coded levels	Actual levels	
	Process components	
	Temperature (°C)	Thickness (mm)
−1	75	2
0	85	3
1	95	4

**TABLE 2:** Experimental runs generated for optimization of drying of jackfruit

Run	A: Temperature (°C)	B: Thickness (mm)
1	70.0	2
2	76.3	4
3	82.5	3
4	95.0	3
5	82.5	4
6	76.3	2
7	95.0	2
8	82.5	3
9	88.8	2
10	82.5	3
11	95.0	2
12	95.0	4
13	82.5	3
14	95.0	4
15	70.0	3
16	70.0	4
17	82.5	3
18	70.0	2
19	82.5	2
20	70.0	3
21	95.0	3

### 2.2 Jackfruit preparation

Ripe jackfruit procured from Kayunga district, Uganda, was used for pulp preparation. The fruits were washed using tap water to remove foreign matter from the surface. The cleaned fruits were peeled, and the arils and seeds separated. The arils were crushed, intermittently for 1 min, in a food blender (Living food diet, Manila) to produce a pulp.

### 2.3 Refractance window drying

Drying was done using a hybrid batch scale refractance window dryer (Utility Model reference number UG/U/2020/000012) on a Mylar sheet (k‐mac plastics‐Type D clear, thickness 0.010 inches). Electricity was used as a source of heat energy to power the drying system. Uniform thickness of jackfruit pulp was achieved using a specially fabricated slate that spread the puree on the top surface of the Mylar float. A humidity and temperature data logger (Extech RT 30, Extech Instruments, USA) was used to ascertain the completion of drying. A hot air oven was used to determine residual moisture in jackfruit leather after completing the drying process.

### 2.4 Analytical methods

#### 2.4.1 Ascorbic acid

Determination of ascorbic acid was done using 2,6‐dichloroindophenol (DCPIP) according to the AOAC method 967.2 (Nielsen, 2019). Five grams of jackfruit pulp was weighed, and ascorbic acid was extracted using an extracting solvent (prepared by mixing 400 ml of glacial acetic acid and 89 ml of orthophosphoric acid and the mixture made to 2.5 L) using a mortar and pestle. The extract was transferred into a 50‐mL volumetric flask to which extracting solvent was added up to the mark. The mixture was shaken vigorously, and 5 ml pipetted out into conical flasks. The solution was titrated against standard DCPIP until a gross pink color was stable for 1 min. The ascorbic acid content was then obtained by calculation using the formula below.

(1) $$\mathrm{Ascorbic}\;\mathrm{acid}\;\left(\mathrm{mg}/100\;\mathrm{ml}\right)=\frac{\mathrm{Net}\;\mathrm{titer}\;\times \;\mathrm{Conc}.\;\mathrm{of}\;\mathrm{DCPIP}\;\times \;\mathrm{Total}\;\mathrm{volume}\;\times \;100\;}{\mathrm{Volume}\;\mathrm{pipetted}\;\times \;\mathrm{Sample}\;\mathrm{weight}}$$

#### 2.4.2 Total antioxidant activity

Two grams of jackfruit pulp was extracted using a 10 ml extraction solution (80% aqueous methanol) and placed in falcon tubes. The falcon tubes containing the mixture were suspended in ultrasonic water (Bransonic series, M.2800‐E; Branson Ultrasonics, Co, Danbury, CT, USA) and subjected to ultrasonic treatment for approximately 20 min at room temperature to allow extraction to take place. The extracts were then cooled for 10 min in a freezer at 4°C and, after that, centrifuged at 3,000 *g* for 10 min (Fisher Scientific 225, Fisher Scientific Co, St Louis, MO, USA). The supernatant was removed from the mixture and collected into a separate tube. The remaining mixtures were further re‐extracted under the conditions previously described to ensure efficient extraction. The two supernatants were mixed and the mixture used for further analysis.

The total antioxidant activity of the methanol extracts was estimated using the 1, 1‐diphenyl‐2‐pycrylhydrazyl (DPPH)‐free radical scavenging assay (Kim et al., 2002). To a 2.95 ml solution of freshly prepared 80% methanol solution, 100 μM solution of DPPH and 50 μl of the sample extract or control (80% [v/v] methanol) were added. The mixture was shaken and allowed to stand in the dark at room temperature (23.7 ± 2°C) for 30 min. The absorbance of the resulting solution was measured at 517 nm in a GENESYS spectrophotometer 10 ultraviolet (Thermo Electron Corporation, Madison, WI, USA) against a blank solution of 80% methanol. The free radical scavenging activity was calculated as follows:

(2) $$\text{Scavenging activity (\textbackslash{}\%)}=\left[1‐\left(\frac{\text{absorbance of sample}}{\text{absorbance of sample}}\right)\right]\times 100$$

A standard solution of ascorbic acid was run using several concentrations ranging from 0.01 to 0.1 mg/ml. A standard calibration curve of ascorbic acid was then prepared by plotting the percentage of free radical scavenging activity against concentration (*R* ^2^ = 0.9981). The final value was expressed as milligram ascorbic acid equivalents per 100 g.

#### 2.4.3 Total carotenoids

The total carotenoid content of the jackfruit pulp was determined according to the method described by Rodriguez‐Amaya and Kimura (2004) with some modifications. A sample (1 g) was ground with 50 ml of acetone, and the decanted liquid was filtered in a 50‐ml volumetric flask using glass wool. The sample was ground until the extract was colorless and no more color could be obtained from the sample. The filtrate was transferred to a 250 ml separating funnel to which 30 ml of petroleum ether was already added. Approximately 250 ml of distilled water was added slowly to the mixture, letting it flow along the walls of the funnel. The two phases separated, and the aqueous (lower) phase was discarded. The upper phase was washed four times with distilled water (250 ml each time) to remove any residual acetone. In the last washing, the lower phase was discarded as completely as possible, without discarding any of the upper phases. The petroleum ether phase was then collected in a volumetric flask (50 ml) while being passed through a small funnel containing anhydrous sodium sulfate (10 g) to remove residual water. The separatory funnel was washed with petroleum ether, collecting the washings in the volumetric flask while passing through the funnel with sodium sulfate. The solution was made up to the 50 ml mark using petroleum ether. The absorbance of the sample was taken at 450 nm using a Spectrophotometer (Spectroquant^®^ Pharo 300, EU), and the total carotenoid content was calculated using the formula below. The experiment was carried out in triplicates.

(3) $$\mathrm{Total}\;\mathrm{carotenoids}\;\left(\mu g/g\right)=\frac{\mathrm{Absorbance}\;\times \;\mathrm{Total}\;\mathrm{volume}\;\times \;10^{‐4}}{\mathrm{Sample}\;\mathrm{weight}\;\times \;2592}$$

### 2.5 Optimization of drying conditions

The responses measured as described above were individually expressed as functions of the independent variables. The data were fitted on the cubic polynomial model using Equation 4 below.

(4) $$\begin{array}{c} Y=b_{0}+\sum_{A=1}^{A=k}b_{1}A+\sum_{B=1}^{B=j}b_{2}B+\sum_{A=1,B=1}^{A=k,B=j}b_{3}AB+\sum_{A=1}^{A=k}b_{4}A^{2}\\ +\sum_{B=1}^{B=j}b_{5}B^{2}+\sum_{A=1,B=1}^{A=k,B=j}b_{6}A^{2}B+\sum_{A=1,B=1}^{A=k,B=j}b_{7}AB^{2}\\ +\sum_{A=1,B=1}^{A=k,B=j}b_{8}A^{2}B^{2}+\sum_{A=1}^{A=k}b_{9}A^{3}+\sum_{B=1}^{B=j}b_{10}B^{3}+\varepsilon \end{array}$$

where *Y* is the response function (drying time (min), ascorbic acid (mg/100g), antioxidant activity (mg/100 g AA equiv) and total carotenoids (μg/g); ε is the random error; *A* and *B* represent the temperature (°C) and thickness (mm), respectively (independent variables); *b*_o_ represents the value of *Y* when *A_i_* and *B_i_* are equal to zero; *b*_1_, *b*_2_, *b*_3_, *b*_4_, *b*_5_, *b*_6_, *b*_7_, *b*_8,_ *b*_9_, and *b*_10_ represent the coefficients for the linear, quadratic, cubic, and interactive effects; *k* is the upper limit of summation for drying temperature (°C); and *j* is the upper limit of summation for thickness (mm). The significance of the models was determined using model analysis and lack of fit. The desirability function approach (DFA) was then used for optimizing the temperature and time conditions using the numerical method. During desirability determination, ascorbic acid content, antioxidant activity, and total carotenoid content were maximized while drying time was minimized.

#### 2.5.1 Validation of optimum process conditions

Jackfruit was dried using the optimized conditions predicted by the software, and experimental values of the response variables were determined and compared with the theoretical values using *t* test (*p* < .05).

### 2.6 Determination of properties of refractance window dried jackfruit powder

The RW dried jackfruit flakes were ground into powder using a grinder (Philips Model HR1727, Koninklijke Philips N.V). The dried jackfruit was milled and sieved using a 600‐micron sieve (Endecotts, UK). The milled powder was stored in an airtight container before further analyses.

#### 2.6.1 Water solubility index (WSI)

The WSI of the jackfruit powder (JFP) was determined using the method described by Kha et al. (2010). Jackfruit powder (2.5 g) and distilled water (30 ml) were vigorously mixed using a vortex mixer (SI‐100N‐MRC Lab Equipment, UK) in a 50‐ml centrifuge tube for 1 min, incubated at 37°C in a water bath (Grant OLS 200, Grant Instruments, UK) for 30 min, and then centrifuged for 20 min at 11,410 *g* in a Heraeus Megafuge 8 (Thermo Scientific, UK). The supernatant was collected in a preweighed beaker and oven‐dried (MRC DFO‐150, MRC Instruments, UK) at 100 ± 2°C. The WSI (%) was calculated as the percentage of dried supernatant with respect to the amount of the original 2.5 g jackfruit powder.

(5) $$\mathrm{WSI}\;\left(\%\right)=\left(\frac{\mathrm{Dried}\;\mathrm{supernatant}\;\mathrm{weight}}{\mathrm{Initial}\;\mathrm{sample}\;\mathrm{weight}}\right)\times 100$$

#### 2.6.2 Water‐holding capacity

Water‐holding capacity was determined according to Nguyen et al. (2015) with slight modifications. A sample (2.5 g) of JFP was weighed in preweighed 50‐ml plastic centrifuge tubes. For each sample, 10 ml of distilled water was added and well mixed with the sample. Samples were left to stand at room temperature for 30 min. The mixture was centrifuged at 2,852 *g* using a Heraeus Megafuge 8 (Thermo Scientific, UK) for 30 min. After centrifugation, the supernatant was decanted, and the new mass of the sample was recorded. WHC (g water/g of powder) was calculated as shown in Equation 6:

(6) $$\mathrm{WHC}=\;\frac{\mathrm{Total}\;\mathrm{water}\;\mathrm{mass}\;(g)}{\mathrm{Dry}\;\mathrm{matter}\;\mathrm{mass}\;(g)}$$

#### 2.6.3 Oil‐holding capacity

Oil‐holding capacity was calculated according to Nguyen et al. (2015), with slight modifications. Jackfruit powder (2 g) was weighed in a preweighed 50‐ml plastic centrifuge tube. For each sample, 20 ml of refined vegetable oil was added and well mixed using a vortex mixer (SI‐100N‐MRC Lab Equipment, UK) at the highest speed. The samples were allowed to stand at room temperature for 30 min. The sample oil mixture was centrifuged at 2,852 *g* for 30 min, the supernatant was carefully decanted, and the new mass of the sample was recorded.

(7) $$\mathrm{OHC}=\;\frac{\mathrm{Mass}\;\mathrm{of}\;\mathrm{sample}\;\mathrm{including}\;\mathrm{held}\;\mathrm{oil}\;(g)}{\mathrm{Mass}\;\mathrm{of}\;\mathrm{dry}\;\mathrm{material}\;(g)}$$

#### 2.6.4 Bulk density

Bulk density (g/mL) was determined by gently adding 2 g of jackfruit powder into an empty 10‐ml graduated cylinder and holding the cylinder on a vortex mixer (SI‐100N‐MRC Lab Equipment) for 1 min. The bulk density (ratio of the mass of the powder and the volume occupied in the cylinder) was then derived (Kha et al., 2010).

#### 2.6.5 Tapped density

The tapped density of the samples was measured by placing a 2.5 g powder sample in a 10‐ml graduated measuring glass cylinder. The tapped volume was measured after the sample was gently dropped 100 times onto a rubber mat from a height of 15 cm. Subsequently, the tapped density was calculated by dividing the weight of the powder by the tapped volume (Kha et al., 2010).

#### 2.6.6 Powder flow properties

##### Hausner ratio and Carr index

The Carr index and the Hausner ratio were used to investigate the flow property of the JFP sample. The Carr index and the Hausner ratio were calculated from the bulk density and tapped density as shown in Equations 8 and 9 (Saifullah et al., 2016).

(8) $$\mathrm{CI}=\frac{T_{d}‐B_{d}}{T_{d}}\times 100$$

(9) $$\mathrm{HR}=\frac{T_{d}}{B_{d}}$$

where CI is Carr index, *T_d_* is tapped density, *B_d_* is bulk density, and HR is Hausner ratio. Different ranges for the Carr index and the Hausner ratio have been defined by Lebrun et al. (2012), as presented in Table 3.

**TABLE 3:** Flowability classification

Flowability	Carr index (CI), %	Hausner ratio (HR)
Excellent	0–10	1.00–1.11
Good	11–15	1.12–1.18
Fair	16–20	1.19–1.25
Passable	21–25	1.26–1.34
Poor	26–31	1.35–1.45
Very poor	32–37	1.46–1.59
Very very poor	>38	>1.60

### 2.7 Cookie formulation

JFP was mixed with wheat flour at two levels (25% and 50%) to prepare cookies (Hosamani, 2016). One sample made without the inclusion of JFP served as a control. The cookies were prepared using the formulations in Table 4. Baking was done at 150°C for 25 min. The cooled cookie samples were kept in sealed containers.

**TABLE 4:** Cookie formulations

Ingredients	Control	25% JF	50% JF
Wheat flour (g)	200	150	100
Jackfruit flour (g)	0	50	100
Sugar (g)	50	50	50
Margarine (g)	50	50	50
Milk (ml)	50	50	50
Eggs	2	2	2
Vanilla (ml)	5	5	5
Baking powder (g)	10	10	10

#### 2.7.1 Textural properties (hardness)

The hardness of the cookies was measured using a Texture Analyser (TA‐XTplus, Stable Micro Systems, Godalming, UK). The texture of cookies was determined with a 3‐point bending rig, 5 kg load cell) and the pretest, test, and post‐test speeds were 1.5, 2, and 10 mm/s, respectively, with a 3 mm compression distance. Hardness (as the fracture force) of cookies was set at a trigger force of 5.0 g using a load cell of 30 kg. The cookie hardness was determined by maximum force (*N*) during compression. Tests were done in quadruplicate.

#### 2.7.2 Sensory evaluation

Fifty semitrained panelists assessed coded samples of cookies (Hough et al., 2006). Each panelist received three cookies from each formulation (control, 25% JFP, 50% JFP). Random three‐digit numbers were used to code the cookies. The sensory acceptance of the cookies was assessed using a 9‐point Hedonic scale (color, taste, mouthfeel, aftertaste, aroma, and overall acceptability), where 1 = dislike extremely and 9 = like extremely.

#### 2.7.3 Ascorbic acid determination of cookies

Jackfruit cookies were ground into a powder using a mortar and pestle. Jackfruit cookie powder (0.5 g) was weighed, and ascorbic acid determination was done using the method outlined in Section 2.4.1.

### 2.8 Statistical analysis

Response surface methodology (RSM) was performed, using Design‐Expert software 2018 version 12, for optimization of independent process parameters, viz., drying temperature (°C) and pulp thickness (mm). The significance of model terms was determined using analysis of variance (ANOVA). The lack of fit and coefficient of determination *R* ^2^ analyses were done to test the adequacy of the developed model. Results obtained from other analyses were subjected to statistical analysis of variance (ANOVA) using XLSTAT software version 2019 to determine variation between means. Duncan's test was conducted to analyze differences between means at a 95% confidence interval.

## 3 RESULTS AND DISCUSSION

Drying time of the pulp for the different conditions varied from 40 to 135 min while ascorbic acid, antioxidant activity and total carotenoids content of the dried pulp varied from 56.20 to 106.00 mg/100 g, 45.90 to 82.60 mg ascorbic acid equivalents/100g and 6.98 to 22.1 µg/g, respectively.

### 3.1 Effect of process variables on drying time of jackfruit pulp

**FIGURE 1:**
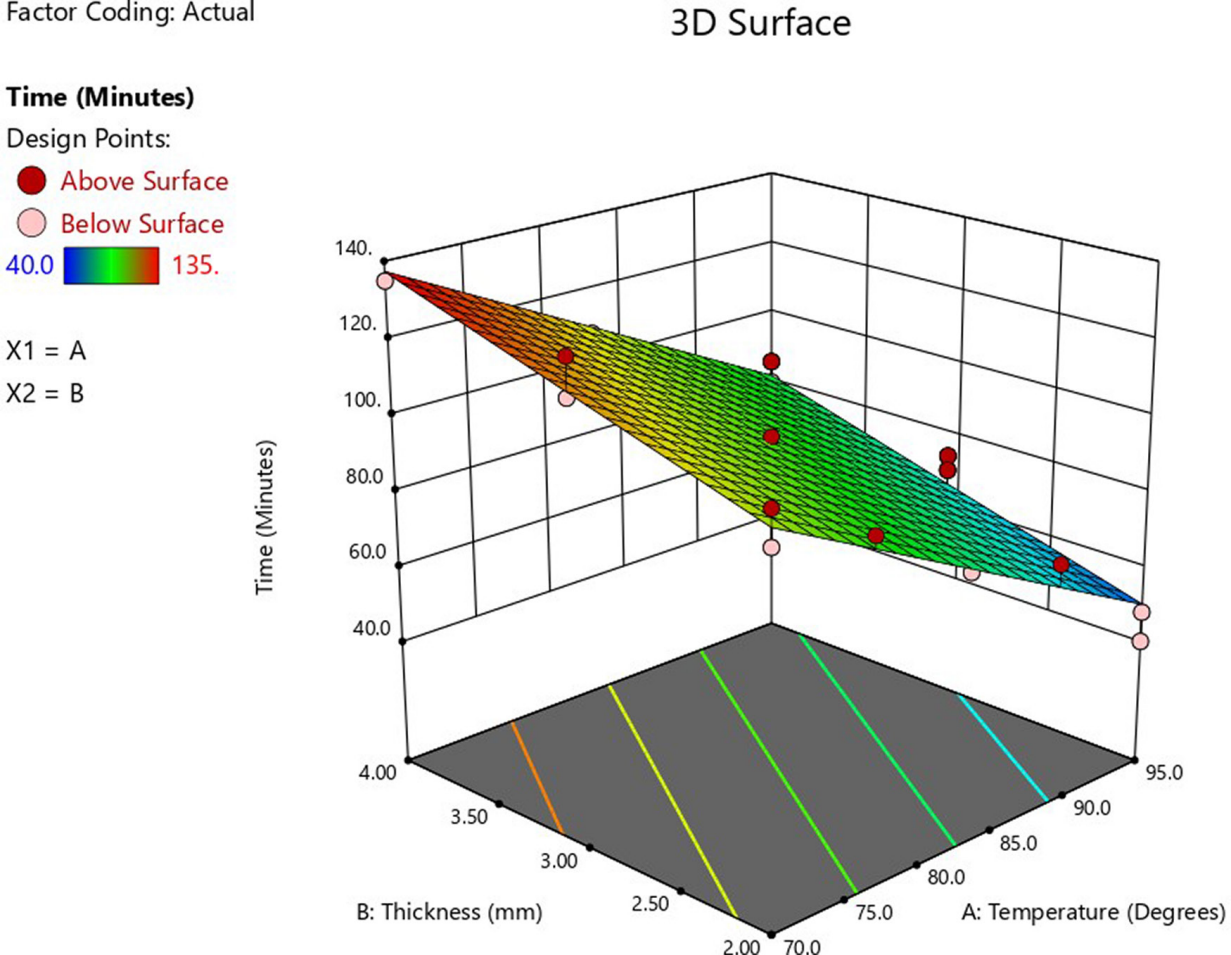
Response surface plot showing the effect of drying temperature and pulp thickness conditions on drying time

An increase in thickness resulted in an increase in drying time, whereas drying time decreased with the increase in drying temperature (Figure 1). Drying takes a shorter time in thin layers because of the short distance water molecules travel to get extracted (Maskan et al., 2002). On the contrary, increasing the drying temperature increases the evaporation rate, accelerating water removal from the fruit surface fruit (Bahmani et al., 2016). Of the two factors, sample thickness was more influential on drying time. This may be explained by the direct proportionality of the thickness of the sample to the weight of the dry solid (Shende & Datta, 2020). The experimental and theoretical results were in close agreement, as indicated by the proximity between *R* ^2^ (.964) and adjusted *R* ^2^ (.960) values (Table 5). The drying time equation showing the effect of process parameters is given in Table 6.

**TABLE 5:** Statistical evaluation of response variables

Response	Time (min)	Vitamin C (mg/100 g)	Antioxidant activity (mg/100g AA equiv)	Carotenoids (μg/g)
Model *p*‐value	<.0001	3.82E−09	<.0001	<.0001
Lack‐of‐fit *p*‐value	.28	.06	.28	.20
*R* ^2^	.96	0.98	9	.99
Adjusted *R* ^2^	.96	.97	.99	.99
Predicted *R* ^2^	.95	.90	.97	.9

**TABLE 6:** Mathematical models of response parameters

Response	Model equation
Drying time (T)	T = 93.8 – 27.7A + 15.9B
Ascorbic acid (A)	A = 19.2 + 0.0844A − 0.57B − 0.33 AB + 0.514 A^2^ − 4.90 B^2^ + 0.02 A^2^B − 1.60 AB^2^ − 0.814 A^3^ + B^3^
Antioxidant activity (AA)	AA = 77.7 + 41.2A − 10.1B − 3.05AB + 1.58A^2^ − 16.2B^2^ + 11.9A^2^B − 12.6AB^2^ − 43.0A^3^ + B^3^
Total carotenoids (C)	C = 7.44 – 1.11A − 2.80B − 2.57AB + 4.85A^2^ + 3.63B_2_ − 1.25A^2^B − 1.09AB^2^ + 1.17A^2^B^2^

### 3.2 Effect of process variables on ascorbic acid

**FIGURE 2:**
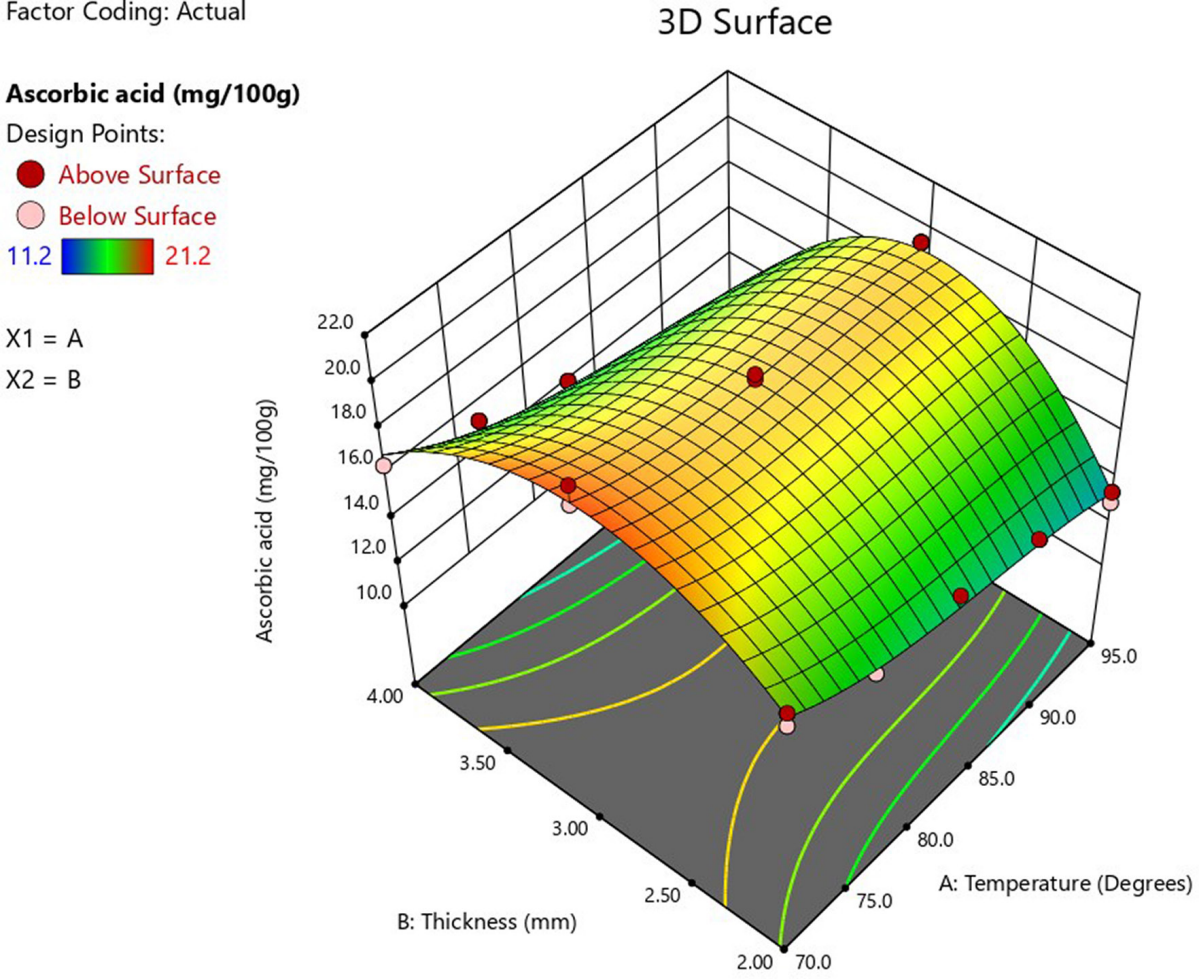
Response surface plot showing the effect of drying temperature and pulp thickness conditions on ascorbic acid

Ascorbic acid degradation occurs during drying due to thermal and aerobic oxidation (Ndawula et al., 2004). The effect of RW drying process parameters on the ascorbic acid content of dried jackfruit leather is shown in Table 5 and Figure 2. The increase in drying temperature resulted in a decrease in ascorbic acid content, attributed to the destruction of ascorbic acid at high temperatures and exposure to oxygen during drying. The conversion rate of ascorbic acid to 2, 3‐diketogulonic acid markedly increases with an increase in temperature, reducing the vitamin C activity (Muzaffar et al., 2016). Ascorbic acid retention was highest for 3‐mm‐thick pulp leather, indicating that extremely thin or thick layers result in more ascorbic acid degradation. For thin layers of pulp, more of the material is exposed to heat and air, resulting in a high level of ascorbic acid oxidation (Nindo et al., 2003; Sogi et al., 2015). For thicker pulp layers, drying takes longer, which means the samples are exposed to heat longer. This is associated with increased loss of ascorbic acid due to the long duration of oxidation (Santos & Silva, 2008). The equation showing the effect of the process parameters is given in Table 6.

### 3.3 Effect of process variables on antioxidant activity

**FIGURE 3:**
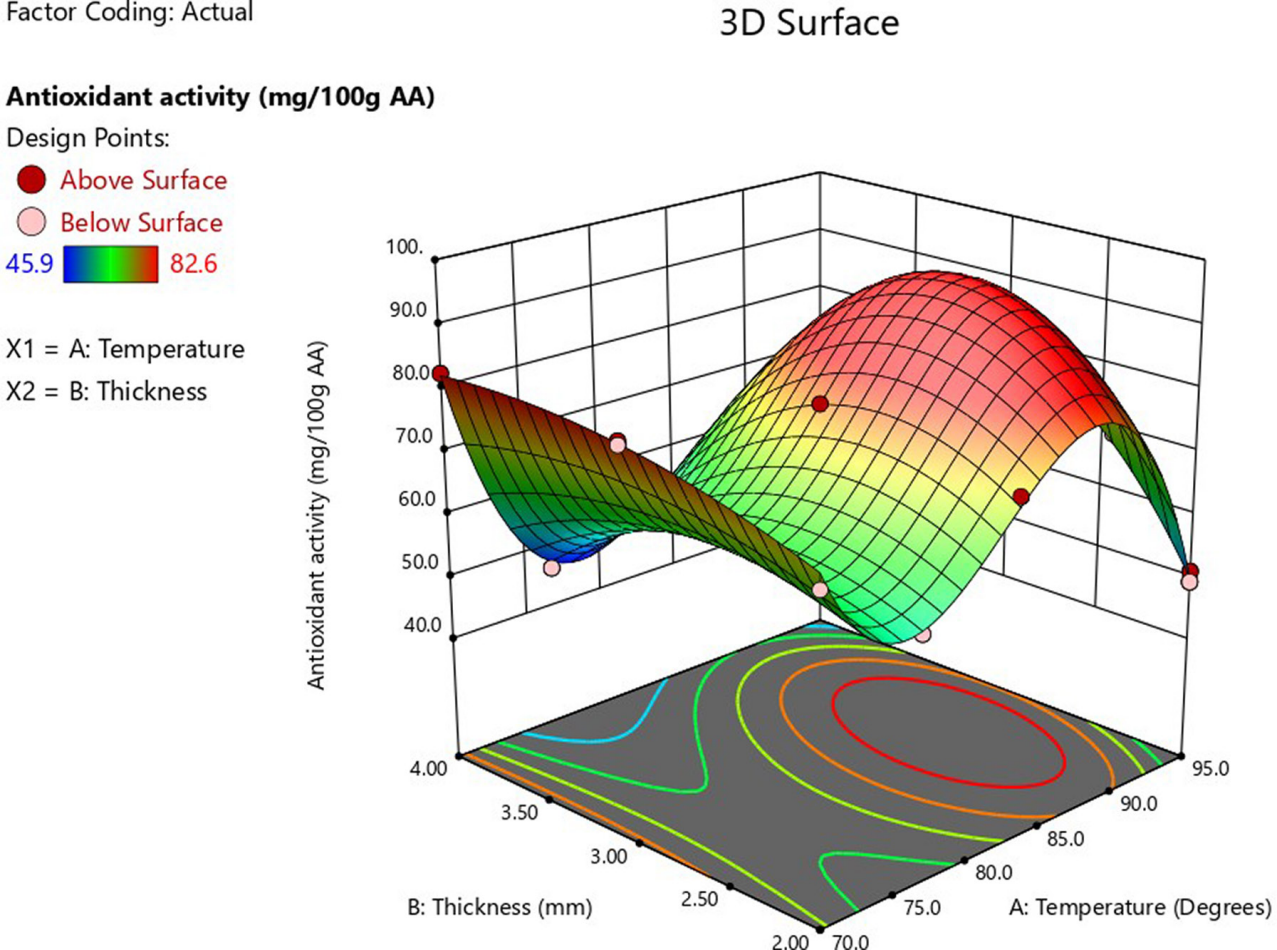
Response surface plot showing the effect of drying temperature and pulp thickness conditions on antioxidant activity

There was an increase in antioxidant activity with an increase in drying temperature, while an increase in thickness decreased antioxidant activity (Figure 3). These results agree with Madrau et al. (2009), who reported a significant increase in the antioxidant capacity of cafona apricot with the increase in drying temperature. The increased antioxidant activity could be due to a variety of factors, including increased polyphenol antioxidant power at intermediate states of oxidation, increased reducing sugar, and formation of Maillard reaction products, which are known to have significant antioxidant activity and are often exerted in a chain‐breaking and DPPH type mechanism (Manzocco et al., 2000). The effect of interactions was observed with a significant model and a nonsignificant lack of fit (Table 5). The equation showing the effect of the process parameters is given in Table 6.

### 3.4 Effect of process variables on total carotenoids content

**FIGURE 4:**
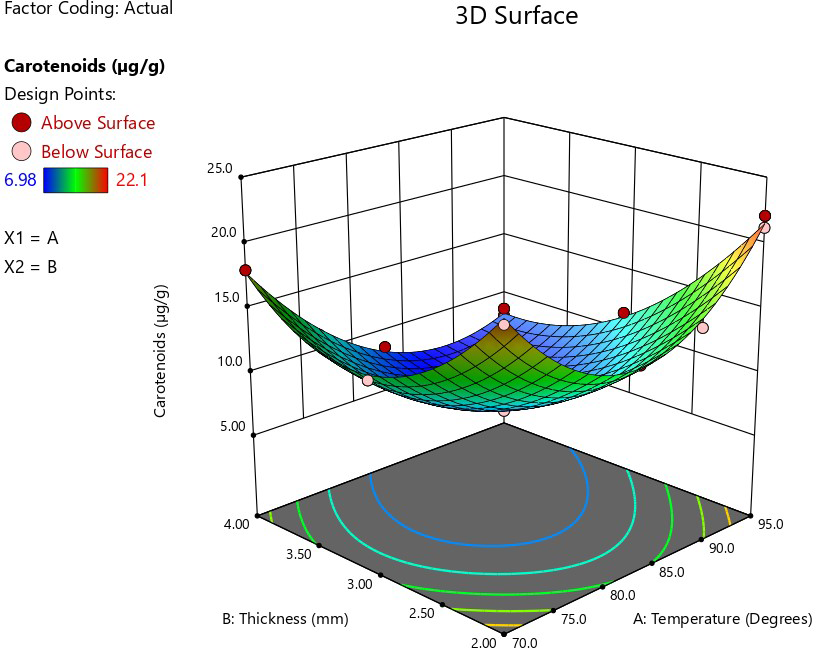
Response surface plot showing the effect of drying temperature and pulp thickness conditions on carotenoid content

An increase in temperature and thickness had a negative impact on total carotenoid content (Figure 4). Total carotenoids are destroyed by heat, light, and oxygen or a combination of all three. Increasing the surface area of the jackfruit by pulping and drying exposes carotenoids in the food to degradation. Consequently, the processing of plant foods is often associated with a decrease in the amount of carotenoids. The longer the processing time, the more effect it causes on the carotenoid content. According to Rodriguez‐Amaya and Kimura (2004), reducing processing time; lowering the temperature; and shortening the time between peeling, cutting, or puréeing and processing improve carotenoid retention dramatically. Rapid processing at high temperatures is a good alternative. The effect of interactions was observed with a significant model and a nonsignificant lack of fit (Table 5). The equation showing the effect of the process parameters is given in Table 6.

### 3.5 Optimization

A numerical multiresponse optimization technique of Design‐Expert^®^12 statistical software (Stat‐Ease, Inc., Minneapolis, USA) was used to determine the optimum conditions for drying jackfruit. The importance of the response variables, including ascorbic acid, antioxidant activity and carotenoids, was set at 3, 3, and 2, respectively. Optimization was applied to the selected ranges of temperature and thickness, and the optimum level of the independent variables with predicted values of the responses was generated by the software. A desirability value of 0.719 was selected with a drying time of 93.4°C and thickness of 2.56 mm as the most desirable solutions for the optimum refractance window drying operating conditions. Under these optimized conditions, the predicted values for ascorbic acid, total carotenoid, and total antioxidant activity were 18.2 mg/100 g, 13.5 μg/g, and 82.6 mg/100 g ascorbic acid equiv.

### 3.6 Validation of optimum process parameters

There was no significant difference (*p* < .05) between the predicted values under the optimized conditions and the experimental values for all the response variables (Table 7). This indicates the suitability of the model in predicting the RWD operating conditions to produce jackfruit leather.

**TABLE 7:** Results obtained in the validation of the conditions optimized for jackfruit leather

	Drying time (min)	Ascorbic acid (mg/100 g)	Antioxidant activity (mg/100 g AA equiv)	Total carotenoids (μg/g)
Observed values	60.47 ^a^ ± 4.34^1^	17.97^a^ ± 2.18^1^	82.34 ^a^± 0.45^1^	13.34 ± 0.29^1^
Optimum predicted model	62.6^a^	18.2^a^	82.6^a^	13.5^a^

^1^ Presented data are at 95% confidence interval.

^a^ No significant difference between the observed values and the predicted values.

### 3.7 Properties of jackfruit powder

The physical properties of fruit powders influence the design, optimization, and performance of fruit powder products. Table 8 shows the assessed material properties of JFP compared with other fruit powders. The bulk density of JFP was comparable to that of pineapple powder (Saifullah et al., 2016). The higher the powder density, the lower the porosity. Porosity is important from the point of dissolution of any powdery material. A less porous material may result in a low dissolution rate (Saifullah et al., 2016). Jackfruit powder demonstrated excellent flowability compared with mango and pineapple fruit powders. Lu et al. (2017) found a strong correlation between powder flowability and dispersion performance. WHC and OHC are important functional properties that are useful for understanding the physiological effects of dietary fiber (Lian & Chong, 2015). The WHC of jackfruit powder was lower than the values reported for mango and pineapple powders (Saifullah et al., 2016). JFP also had notably lower OHC compared with mango but similar to values for pineapple powder. Differences in the properties of the different powders could be attributed to differences in their chemical and physical structure and polysaccharides.

**TABLE 8:** Jackfruit powder properties

Properties	Jackfruit powder *Experimental values*	Mango powder^a^	Pineapple powder^a^
Bulk density (g/cm^3^)	0.591 ± 0.00	0.638 ± 0.48	0.579 ± 0.39
Tapped density (g/cm^3^)	0.596 ± 0.00	0.833 ± 0.11	0.749 ± 0.42
Carr index	0.871 ± 0.24	23.430.24	22.65 ± 0.21
Hausner ratio	1.008 ± 0.00	1.31 ± 0.17	1.29 ± 0.12
Flowability	Excellent	Passable	Passable
Water holding capacity (g/g)	1.153 ± 0.02	6.4	14.6
Oil holding capacity (g/g)	0.847 ± 0.03	1.6	0.7
Solubility index (%)	73.22 ± 1.04	ND	ND

Abbreviation: ND, Not determined

^a^ Saifullah et al. (2016).

### 3.8 Sensory evaluation, textural, and ascorbic acid content of jackfruit cookies

The substitution of wheat flour with jackfruit powder did not affect the texture of the cookies, which is a critical kinesthetic characteristic. Overall acceptability combines all the attributes covering individual judgment by the panelists. The sensory evaluation results obtained (Table 9) showed that 25% JFP‐incorporated cookies had a higher mean score for all attributes than the control and 50% JFP‐incorporated cookies. Similarly, Hosamani (2016) established that jackfruit biscuits with 25% jackfruit powder had the highest score (4.58 out of five, on a five‐point hedonic scale) for overall acceptability. Biscuits with 50% jackfruit powder added had the lowest (3.78 out of five, on a five‐point hedonic scale), similar to the results obtained in this study. Hosamani (2016) hypothesized that this could be due to unattractive color and unpalatable taste and flavor at higher proportions of jackfruit powder. According to Everitt (2009), a mean liking score of 7 or higher on a nine‐point scale usually indicates highly acceptable sensory quality; hence, a product achieving this score could be used confidently as a good illustration of “target” quality. Based on the sensory acceptability results, 50% JFP‐incorporated cookies were therefore well accepted by panelists.

**TABLE 9:** Hardness, sensory properties, and ascorbic acid content of jackfruit cookies

Attribute	0% JFP	25% JFP	50% JFP
Color	6.460 ± 1.91^a^	7.860 ± 1.05^b^	6.140 ± 2.17^a^
Aroma	6.960 ± 1.28^a,b^	7.060 ± 1.50^b^	6.300 ± 2.39^a^
Taste	6.720 ± 1.63^a^	7.500 ± 1.16^b^	6.560 ± 1.92^a^
Mouthfeel	6.560 ± 1.58^a^	7.320 ± 1.25^b^	6.200 ± 2.05^a^
After‐taste	6.420 ± 1.91^a,b^	7.020 ± 1.71^b^	6.080 ± 2.25^a^
Overall acceptability	6.820 ± 1.35^a^	7.540 ± 1.15^b^	6.380 ± 1.98^a^
Maximum force *N*	26.067 ± 3.37^a^	30.723 ± 6.84^a^	24.196 ± 4.32^a^
Ascorbic acid (mg/100g)	4.970 ± 0.00^a^	11.73 ± 0.65^b^	19.57 ± 0.28^c^

Note

Values of different letters within the same column are statically different from each other (*p* < .05); presented data are mean value ± standard deviation (*n* = 50).

Beneficial effects of the addition of jackfruit powder included an increased ascorbic acid content of the cookies. Cookies with 50% jackfruit powder had the highest vitamin C content, followed by cookies with 25% jackfruit powder. The addition of jackfruit powder increased ascorbic acid content by 32.9% in 25% JFP enriched cookies and 66.8% in 50% JFP enriched cookies.

## 4 CONCLUSIONS

Optimization of RW drying process parameters was successfully done by using response surface methodology (RSM). Twenty‐one experiments were conducted in triplicates to obtain optimum drying conditions of 93.4°C drying temperature and 2.56 mm pulp thickness. The optimal RW drying time for jackfruit pulp was short (60 min) compared with solar drying (3 days) and oven drying (18 h), and the resultant powder was rich in ascorbic acid. Jackfruit powder formulated cookies, up to 25% were well accepted by their sensory characteristics. The incorporation of RWD jackfruit powder into cookies also significantly increased their ascorbic acid content. So, the use of jackfruit powder flour in cookies was effective for technological and nutritional advantages. The findings confirm the potential to utilize jackfruit powder to produce nutrient enhanced baked products to improve nutrient intake.

## CONFLICT OF INTEREST

The authors declare that there is no conflict of interest.

## Data Availability

The data sets used or analyzed during the current study are available from the corresponding author on reasonable request.
